# Putative Protein Biomarkers of *Escherichia coli* Antibiotic Multiresistance Identified by MALDI Mass Spectrometry

**DOI:** 10.3390/biology9030056

**Published:** 2020-03-19

**Authors:** Telma de Sousa, Didier Viala, Laetitia Théron, Christophe Chambon, Michel Hébraud, Patricia Poeta, Gilberto Igrejas

**Affiliations:** 1 Department of Genetics and Biotechnology, University of Trás-os-Montes and Alto Douro (UTAD), 5000-801 Vila Real, Portugal; telmaslsousa@hotmail.com; 2 Microbiology and Antibiotic Resistance Team (MicroART), Department of Veterinary Sciences, University of Trás-os-Montes and Alto Douro (UTAD), 5000-801 Vila Real, Portugal; ppoeta@utad.pt; 3 Functional Genomics and Proteomics Unit, University of Trás-os-Montes and Alto Douro (UTAD), 5000-801 Vila Real, Portugal; 4 Associate Laboratory for Green Chemistry (LAQV), Chemistry Department, Faculty of Science and Technology, University Nova of Lisbon, Lisbon, 2829-516 Caparica, Portugal; 5 Institut National de Recherche pour l’Agriculture, l’Alimentation et l’Environnement (INRAE), Metabolomic and Proteomic Exploration Facility (PFEMcp), 63122 Saint-Genès-Champanelle, France; didier.viala@inrae.fr (D.V.); christophe.chambon@inrae.fr (C.C.); michel.hebraud@inrae.fr (M.H.); 6 Institut National de Recherche pour l’Agriculture, l’Alimentation et l’Environnement (INRAE), UR Qualité des Produits Animaux (QuaPA), 63122 Saint-Genès-Champanelle, France; laetitia.theron@inrae.fr; 7 Université Clermont Auvergne, INRAE, UMR Microbiologie Environnement Digestif Santé (MEDiS), 63122 Saint-Genès-Champanelle, France

**Keywords:** antibiotics, bacterial resistance, one health, MALDI-TOF MS, biomarkers

## Abstract

The commensal bacteria *Escherichia coli* causes several intestinal and extra-intestinal diseases, since it has virulence factors that interfere in important cellular processes. These bacteria also have a great capacity to spread the resistance genes, sometimes to phylogenetically distant bacteria, which poses an additional threat to public health worldwide. Here, we aimed to use the analytical potential of MALDI-TOF mass spectrometry (MS) to characterize *E. coli* isolates and identify proteins associated closely with antibiotic resistance. Thirty strains of extended-spectrum beta-lactamase producing *E. coli* were sampled from various animals. The phenotypes of antibiotic resistance were determined according to Clinical and Laboratory Standards Institute (CLSI) methods, and they showed that all bacterial isolates were multi-resistant to trimethoprim-sulfamethoxazole, tetracycline, and ampicillin. To identify peptides characteristic of resistance to particular antibiotics, each strain was grown in the presence or absence of the different antibiotics, and then proteins were extracted from the cells. The protein fingerprints of the samples were determined by MALDI-TOF MS in linear mode over a mass range of 2 to 20 kDa. The spectra obtained were compared by using the ClinProTools bioinformatics software, using three machine learning classification algorithms. A putative species biomarker was also detected at a peak *m*/*z* of 4528.00.

## 1. Introduction

It is estimated that 18% of the genes present in the genome of *Escherichia coli*, a commensal bacteria in humans and other animals, come from horizontal transfer from other species over millions of years [1]. Normally, these bacteria are not responsible for the onset of diseases, but when they acquire virulence factors, they can cause severe infections. As an additional consequence, these bacteria have a high capacity to propagate and pass these genes on even to phylogenetically distant bacteria [2,3]. With the emergence of new resistances, there has thus been a decrease in the effectiveness of treatments for certain bacterial infections in human and veterinary medicine, causing serious problems worldwide [4].

Antibiotics act in several different ways, such as by inhibiting cell wall biosynthesis, altering cell membrane permeability, interfering in replication and repair of DNA, inhibiting protein synthesis, and inhibiting the biosynthesis of folic acid. The mechanism of action of each antibiotic prevents one or more crucial steps in the cellular processes of the target microorganism essential for its survival [5,6]. The inappropriate use of antibiotics can lead to serious problems, especially in some developing countries where there are few health technicians and antibiotics are commonly available for purchase without prescription [7]. Even when the prescription of antibiotics is adequate, the dosage regimens may be too short to eradicate the infection, intensifying the survival of resistant strains [7]. These factors are contributing to the emergence of new and potentially hazardous bacterial strains.

In 1975, Anhalt and Fenselau pioneered the use of mass spectrometry (MS) for the identification of bacteria, and it has proved a powerful tool for identifying the species and the biomarkers of certain diseases [8]. It is also a way of gaining deeper insights into cell events by pinpointing the proteins involved in different cellular processes. Proteomics is now an extremely important way of studying the differential expression of proteins under different environmental conditions, and is leading to the discovery of new biomarkers that are more or less abundant in a state of disease compared to a healthy state [9,10]. This technique has been demonstrated to be useful for studying antibiotic resistance in different bacterial strains, especially when determining the metabolic pathways involved [11]. As multi-antibiotic resistant bacteria can hinder the treatment of patients with complex infections, rapid and effective detection of resistance mechanisms may be vital in choosing the best antimicrobial to use. While some proteins related to different antimicrobial resistance mechanisms have been identified, the need to discover new biomarkers has been highlighted [11,12]. This task is challenging because it involves multidisciplinary skills, requiring the collaboration of scientists expert in genetics, molecular biology, biochemistry, mass spectrometry, and bioinformatics [9]. When *E. coli* biomarkers were characterized by MALDI-TOF MS, most of the biomolecules detected were ribosomal proteins. Many ribosomal proteins are found in the cell envelope subfraction, because they are often located just inside the cell membrane of intact bacteria [13,14]. As biomarkers are discovered, comprehensive information on the nature of proteins and their expression in relation to antimicrobial agents and resistance mechanisms must be stored in curated databases [15].

For this study, the principal aims were to characterize isolates of *E. coli* by MALDI-TOF MS and then, based on genotypic characterization, to identify and analyze at a statistical level the protein biomarkers associated with the antibiotic profiles of the bacteria and their responses to antibiotics.

## 2. Materials and Methods

### 2.1. Samples and Bacterial Strains

The majority of the *E. coli* strains used in this work came from animals and were collected in several regions of Portugal and Spain over different periods of time (Table 1). A few strains were collected from human patients at the Gabriel-Montpied University Hospital Centre in Clermont-Ferrand (France). To isolate individual strains, the samples were spread on Petri dishes containing Levine agar supplemented with cefotaxime (2 mg/mL). After 24 h of incubation at 37 °C, colonies typical of *E. coli* were selected, purified, and identified by standard bacteriological methods (i.e., Gram-staining, catalase, oxidase, indol, methyl-red/Voges-Proskauer, citrate, and urease), and by using the API 20E biochemical identification system (bioMérieux, La Balme Les Grottes, France).

### 2.2. Antimicrobial Susceptibility Test and Characterization of Resistance Genes

Thirty recovered *E. coli* isolates were selected at random and tested using the agar disk diffusion method, as recommended by the Clinical and Laboratory Standards Institute (CLSI) [16]. The antimicrobials tested were streptomycin (STR; 10 μg/disk), tetracycline (TET; 30 μg/disk), gentamicin (CN; 10 μg/disk), tobramycin (TOB; 10 μg/disk), kanamycin (K; 30 μg/disk), ciprofloxacin (CIP; 5 μg/disk), trimethoprim–sulfamethoxazole (SXT; 1.25/23.75 μg/disk), cefoxitin (FOX; 30 μg/disk), and chloramphenicol (CHL; 30 μg/disk). The following antimicrobial resistance genes were studied by PCR, as previously described [17,18,19,20]: *tet*(A) and *tet*(B) in TET-resistant isolates; *aadA* and *strA-strB* in STR-resistant isolates; *aac(3)-II* and *aac(3)-IV* in CN-resistant isolates; *sul1*, *sul2*, and *sul3* in SXT-resistant isolates; *cmlA* in CHL resistant isolates; *bla*_CTX-M_, *bla*_TEM_ and *bla*_SHV_ in AMP-resistant isolates (alleles for *bla*_CTX-M_ were obtained by sequencing in a previous studies); and *ampC* in FOX-resistant isolates.

### 2.3. Proteomics Study

#### 2.3.1. Bacterial Culture with and without Antibiotics

From all the *E. coli* isolates obtained, 30 were randomly selected (*n* = 30) and seeded in 5 mL of brain heart infusion liquid (BHI) at 37 °C for 24 h to promote bacterial growth and ensure that the bacterial concentration was appropriate and equivalent for all the samples. After 24 h, the bacterial density of cultures was determined, and Petri dishes containing 18 mL of Levine medium were inoculated with each strain and incubated at 37 °C for 24 h to check that the bacterial density was appropriate and equivalent for all the samples. The density was determined from the spectrophotometer, where one to two colonies were removed from the Petri dish and diluted in BHI (in order to decrease the bacterial concentration) until the density was below approximately 0.800 (600 nm wavelength).

CLSI standards were followed for the cultures with the different antibiotics tested [16]. Levine agar medium was supplemented with 1 mL of antibiotic at the appropriate concentration. The rest of the culture procedure was the same as for cultures without antibiotic.

#### 2.3.2. MALDI-TOF Mass Spectrometry

Protein extracts were obtained from intact bacterial cells using a quick method described by [21]. For each *E. coli* strain, two colonies were transferred at room temperature to separate solutions containing 300 μL of distilled water and 900 μL of ethanol, then were centrifuged for 2 min at 10,000× *g*. The supernatant was discarded, and the same steps were repeated before drying the pellet at room temperature. The pellet was then vortexed in 10 μL of 70% formic acid until completely dissolved. Then, 10 μL of pure acetonitrile were added, and the sample was centrifuged for 2 min at 10,000× *g* and the supernatant recovered. Supernatant (5 μL) was mixed with 10 μL of matrix solution (10 mg of α-cyano-4-hydroxycinnamic acid in 1 mL of a solution of 50% acetonitrile and 2.5% trifluoroacetic acid).

An aliquot of each sample (1 μL) was deposited on the MALDI-TOF MS target (Anchorchip). Calibration was performed by depositing 1 μL of a mixture containing 0.5 μL of the matrix solution and 0.5 μL of Calibrating Protein Standard I solution. The deposits were left to dry on the MALDI-TOF MS target at room temperature. The sample analyses were carried out with an Autoflex Speed mass spectrometer (Bruker Daltonics) using the following parameters: linear mode, positive-ion extraction with voltages of 19.56 kV at source 1 and 18.09 kV at source 2, time delay about 160 ns, and laser intensity to 90%. For each extracted colony, a spectrum was obtained from the sum of three laser shots at the frequency of 1000 Hz. A calibrant spot was analyzed after each isolate analysis by summing four laser shots at the frequency of 1000 Hz.

#### 2.3.3. Statistics and Bioinformatics

MALDI-TOF MS of the *E. coli* samples were analyzed with the ClinProToolsTM software (version 3.0, Bruker Daltonik). This program can be used to recalibrate the spectrum, calculate the average of each spectrum and the peak statistics, and generate models based on the classification algorithm selected. The ClinProTools software includes four types of machine learning algorithms for generating classification models: Quick Classifier (QC), Genetic Algorithm (GA), Supervised Neural Network (SNN), and Support Vector Machine. The spectra selected were submitted to three of these—QC, GA, and SNN—where cross-validation is based on the accuracy of the algorithm in correctly assigning a random sample to the correct group. For each peak, the receiver operating characteristic (ROC) analysis was performed based on the area under the ROC curve (AUC). The AUC measures discrimination, which is the ability of the test to correctly classify positive and negative instances. If the AUC is close to 1, the test is better.

## 3. Results and Discussion

### 3.1. Phenotypic and Genetic Characterization

**Figure 1:**
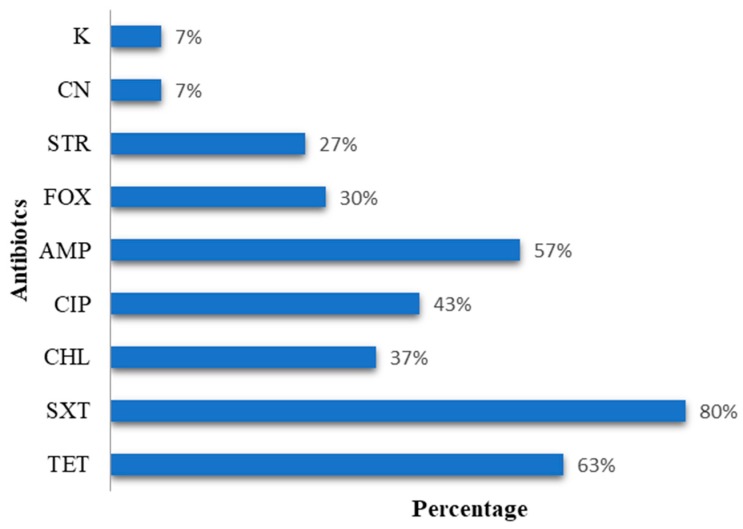
Percentage of phenotypic resistance for *E. coli* to the different antibiotics tested. It can be seen that more than half of the panel of strains are resistant to trimethoprim–sulfamethoxazole, tetracycline, and ampicillin antibiotics (80%, 63%, and 57%, respectively).

By sampling a range of animal and human sources, we detected *E. coli* strains that are highly resistant or resistant to several antibiotics, which is consistent with previous studies [22,23,24]. Among the 30 *E. coli* isolates randomly chosen to be studied in more detail, 26 of them were resistant to at least one antibiotic at the phenotypic level. Most isolates showed resistance to trimethoprim–sulfamethoxazole (*n* = 24), tetracycline (*n* = 19), and ampicillin (*n* = 17). In contrast, only a few isolates were resistant to kanamycin (*n* = 3) and gentamicin (*n* = 2). Figure 1 shows the proportions of the 30 isolates, showing phenotypic resistance to the nine antibiotics tested. In a study of *E. coli* isolates from pigs in Germany, the most common resistance found was against tetracycline in 78.7% isolates [25], which is similar to the frequency reported here in the isolates from pigs, which was 100%. Although the use of chloramphenicol in cattle husbandry was abolished in Europe in the 1990s, a study in the northwest of England shows that strains of *E. coli* continue to have an incidence of resistance to this antibiotic, which is often associated with phenotypes of resistance, possibly as part of a multi-resistance system [26].

The antimicrobial resistance and the presence of antibiotic resistance genes detected in the *E. coli* isolates are detailed in Table 2.

The *tet*(A) gene was found in 13 isolates, while the *tet*(B) gene was only found in five isolates, which might indicate that the *tet*(A) gene is the more prevalent. In contrast, Ahmed et al. showed that the *tet*(B) gene was more prevalent than the *tet*(A) gene. Both *tet*(A) and *tet*(B) genes were found in only two isolates [26].

All the strains encode genes to produce extended-spectrum β-lactamase (ESBL), confirming that these Gram-negative bacteria are resistant to β-lactams. In this study, there was a higher prevalence of *bla*_CTX-M_ (77%) compared to *bla*_SHV_ and *bla*_TEM_ (10% and 40%, respectively). The rapid dissemination of broad spectrum β-lactamases is a global emergency for public health. Most ESBL genes are mutants derived from the β-lactamases *bla*_SHV_ and *bla*_TEM_, but there has been an increasing prevalence of mutations in the gene *bla*_CTX-M_ in Enterobacteriaceae in the last decade [27]. Countries such as Canada, Italy, Spain, Greece, and the United Kingdom have witnessed alarming reports of antimicrobial resistance within ESBL-producing organisms in community locations [28].

Most of the isolates have shown resistance to trimethoprim–sulfamethoxazole and the genes *sul1*, *sul2*, and/or *sul3* have been identified, confirming the phenotypic test. For example, 12 isolates contained the gene *sul2*.

### 3.2. Specific Peak for E. coli

The following MALDI-TOF MS analyses were focused on the proteomics of resistance to trimethoprim-sulfamethoxazole, ciprofloxacin, ampicillin, chloramphenicol and tetracycline, which were the most frequent antibiotic resistances observed in our isolates. For each isolate, the mass spectra were obtained from cells grown with and without the presence of an antibiotic. Most of the peaks obtained from *E. coli* cultures grown in the presence and absence of antibiotics in the medium have a good resolution. Here, the purpose was not to analyze each isolate itself, but to compare two conditions: the first class represents the isolates that grew in the absence of an antibiotic in the culture medium, and the second class contains the isolates that grew in the culture medium with different antibiotics—that is, they were responding to and resisting the antibiotic.

**Figure 2:**
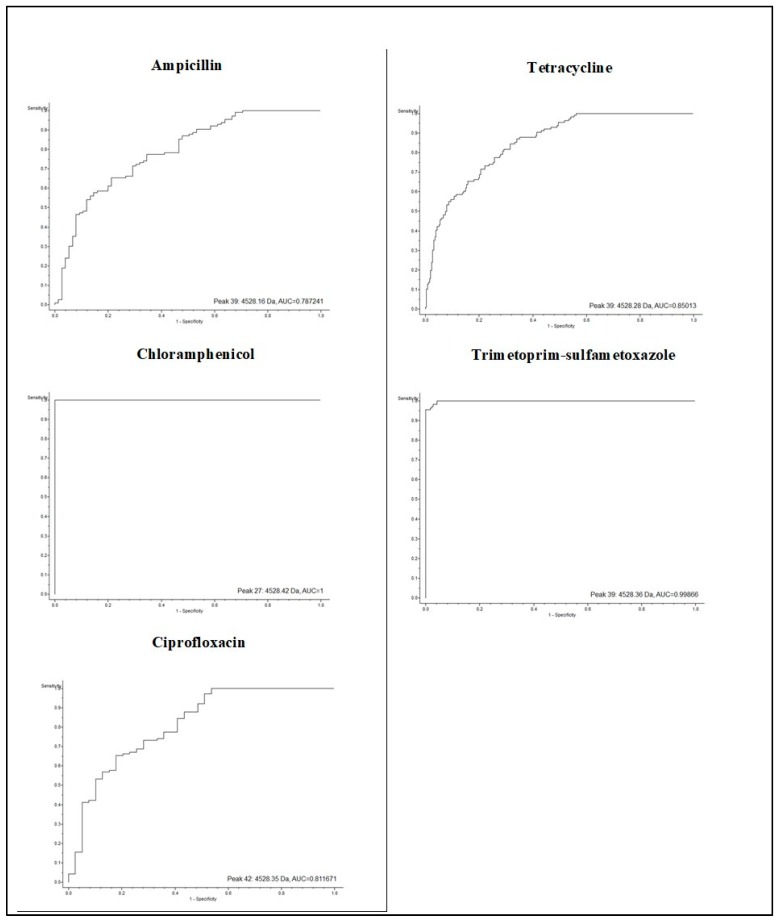
Area under the receiver operating characteristic (ROC) curve (AUC) value for the peak at 4528.42 *m*/*z* obtained from an *E. coli* culture grown in the presence of different antibiotics. The ROC curve allows us to know the sensitivity and specificity of a test, and the area evaluates discrimination, which is the ability of the test to correctly classify positive and negative tests.

For all strains of *E. coli*, a peak *m*/*z* of 4528.00 was detected, and the AUC values were significant (≥0.80), except for those grown in ampicillin, which gave an AUC value of 0.78 (Figure 2). This peak could therefore be considered as characteristic of this bacterial species. On the other hand, another study detected the peaks at 6711.00 *m*/*z* and 10883.00 *m*/*z* were chosen as outbreak *E. coli* strain biomarkers. This variation in the detection of specific peaks may be due to the general spectral variability between endemic strains of *E. coli* [29].

MALDI-TOF MS has performed well in detecting different bacterial species. The peak at 4448.00 *m*/*z* was related to clinical strains of *S. aureus* [30]. The overall correct identification rate of MALDI-TOF MS for anaerobic bacteria ranged from 60% to 100% at the genus level, and from 51% to 100% at the species level [31]. Thus, this technique is demonstrated to have good functionality in the detection of bacterial species and genera in a rapid way.

### 3.3. Specific Peaks for E. coli Strains Grown in Each Antibiotic

#### 3.3.1. Trimethoprim–Sulfamethoxazole and Ampicillin

**Figure 3:**
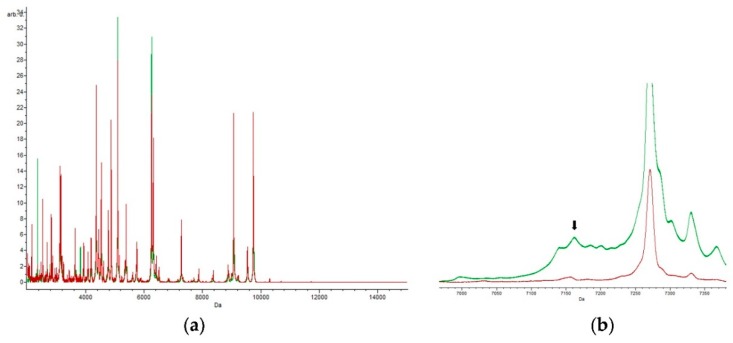
Peaks at 7160.36 *m*/*z*: (**a**) spectrum obtained in the presence of trimethoprim–sulfamethoxazole (green) and (**b**) zooming in on the peak at 7160.36 *m*/*z*, which is statistically significant (*p*-value = 0.000001) and is specific to cells submitted to the trimethoprim-sulfamethoxazole antibiotic.

For *E. coli* strains grown in trimethoprim-sulfamethoxazole, we highlighted 37 peaks with the algorithms Genetic Algorithm, Supervised Neural Network, and Quick Classifier, which gave results of 98.63%, 98.30%, and 90.99%, respectively. These values were also obtained by cross-validation, thus corroborating the models used. The peaks at 3815.82 *m*/*z*, 6112.84 *m*/*z*, and 7160.36 *m*/*z* (Figure 3) have AUC values of 0.83, 0.95, and 0.96, respectively, and only arise from samples of *E. coli* grown with this antibiotic. These peaks may therefore correspond to proteins involved in the trimethoprim–sulfamethoxazole resistance mechanism.

#### 3.3.2. Ampicilin

**Figure 4:**
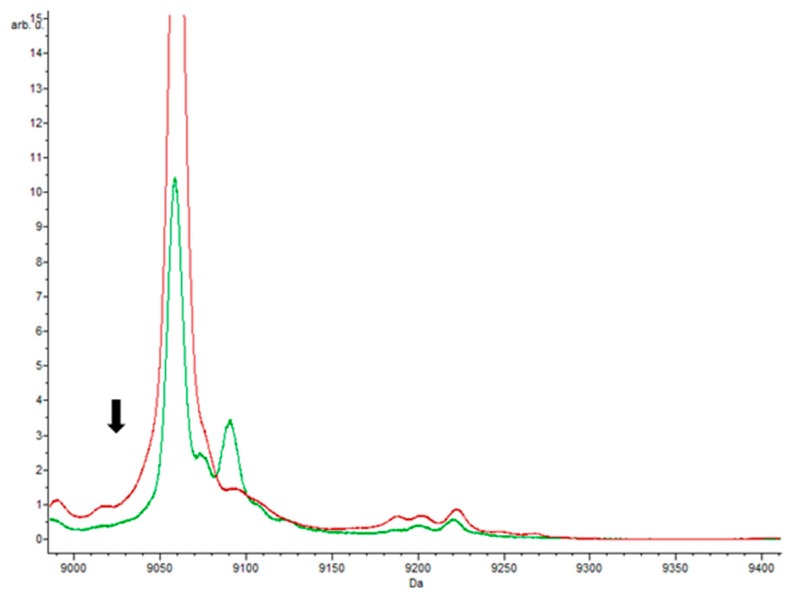
Peaks at 9089.69 *m*/*z*. The characteristic peak obtained with the antibiotic ampicillin (green) vs without antibiotic (red). The *p*-value is 0.0000359, making this peak statistically significant, and is exclusive to ampicillin antibiotic.

The peptide masses 2770.87 *m*/*z*, 2943.29 *m*/*z*, 3665.49 *m*/*z*, 5105.00 *m*/*z*, and 9089.69 *m*/*z* are exclusive to *E. coli* exposed to ampicillin, and are absent from the spectra of control samples. Figure 4 shows the characteristics of the 9089.69 *m*/*z* peak in detail.

The presence of the 2943.29 *m*/*z* peak may reflect the expression of a protein involved in a specific/particular resistance mechanism. A study by Camara and Hays [32] provides additional evidence that the 2943.29 *m*/*z* peak can be detected in *E. coli* samples. The exclusivity of these *m*/*z* values suggests that this could be considered a specific biomarker for this antibiotic response.

In our work, no peak with a peptide mass associated with the SHV-type β-lactamases was found in the ampicillin-resistant isolates, which is confirmed by the genotype, as they only had the *bla_TEM_* and/or *bla_CTX-M_* genes. The genotype shows one sample with the *bla_SHV_* gene; however, its expression was not sufficiently statistically significant for the detection of this kind of β-lactamase.

The family of SHV-type β-lactamases is constantly evolving. Each type of SHV can be distinguished from all other types based on their peptide masses [33,34]. We were thus able to associate the mass peaks at 2009.51 *m*/*z* and 4434.70 *m*/*z* present in the proteomes of the isolates, which were grown in the presence of trimethoprim–sulfamethoxazole, as β-lactamases of the SHV type. This data corroborates the genotype obtained in isolates with resistance to this antibiotic and with the masses obtained in the study by Sturenburg et al. [34]. In this sense, the detection of modifications in this family may become crucial in preventing newly emerged resistances from spreading.

A study developed by Oviaño Marina et al. obtained quite significant positive results in the detection of cefotaxime and ceftazidime hydrolysis for the same incubation time, compared to the use of qualitative spectrum interpretation in ESBL-producing Enterobacteriaceae [35]. However, when comparing the results, there is no direct correlation, since the patterns used in this work (intact proteins) and in the developed work (digested proteins) by Oviaño Marina use different patterns of analysis [35,36]. Nevertheless, the detection of resistance to β-lactamase mediated by β-lactamases in clinical samples by MALDI-TOF MS presented a well-studied pathway with increasingly promising results.

#### 3.3.3. Chloramphenicol

**Figure 5:**
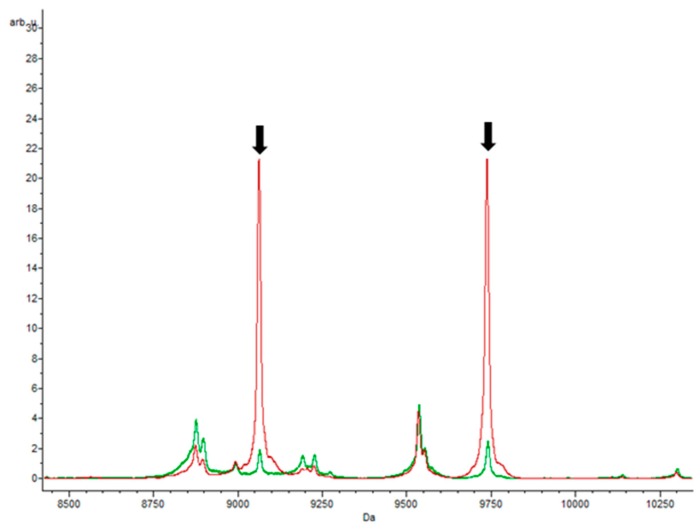
Peaks obtained with chloramphenicol. Spectra obtained in the presence of antibiotic (green) and without antibiotic (red). The results showed statistically significant peaks (*p*-value = 0.000001), with masses of 9059.97 and 9735.29 *m*/*z*, respectively.

Two peaks appeared very significant in the proteomes of *E. coli* grown with chloramphenicol, namely 4866.85 *m*/*z* and 9059.97 *m*/*z*, both of which had AUC values of 0.99. Peaks at 9059.97 and 9735.29 *m*/*z* were both highlighted by the Supervised Neural Network and QuickClassifier algorithms (Figure 5). Although these two peaks also appear in the *E. coli* proteomes responding to other antibiotics, they are much less intense compared to the control samples. Thus, they can also serve as potential biomarkers of the chloramphenicol response through the marked difference in intensity. Conversely, the peaks with masses of 4395.40 *m*/*z*, 5830.14 *m*/*z*, 6350.38 *m*/*z*, and 6367.35 *m*/*z* were only found in the strains that grew in medium with chloramphenicol. Potentially, these peaks could correspond to proteins involved in the mechanism of resistance, since this was the only antibiotic studied that intervenes in the inhibition of protein synthesis through the 50S subunit of the ribosome.

On the other hand, the 9059.97 *m*/*z* peak was found in almost all *E. coli* isolates, which may correspond to the mass of a protein involved in the basal metabolism of the bacterium and is not specific to the cellular response to chloramphenicol. The same peptide mass was found in other *E. coli* strains from humans, cows, and *Shigella flexneri* [37,38]. However, the latter peak was not detected in cells growing in the presence of ciprofloxacin, which raises some questions about how the protein might function.

#### 3.3.4. Tetracycline

Tetracycline was the antibiotic which gave rise to the fewest specific peaks, according to this machine learning approach. Only the 3665.79 *m*/*z* peak was highlighted by two sorting algorithms; other putative peaks were only detected by a single algorithm. Nevertheless, results with cross-validation of model generation were 88.43%, 79.41%, and 75.17% for Genetic Algorithm, Supervised Neural Network, and Quick Classifier, respectively, showing that the peaks were indeed accurately detected even if only by one algorithm. Peaks reported by Sturenburg et al. for the strains exposed to tetracycline were not observed here [34]. These tetracycline-producing streptomyces present important signal and protein spectra in the mass range of 2 to 20 kDa. The mass spectrum of tetracycline (dissolved in ethanol) (M + H +) was found at 445.41 *m*/*z* [39]. This study can be an anchor point for our study, since the detection of specific peaks for tetracycline in our work did not achieve final results when compared to other antibiotics.

#### 3.3.5. Ciprofloxacin

**Figure 6:**
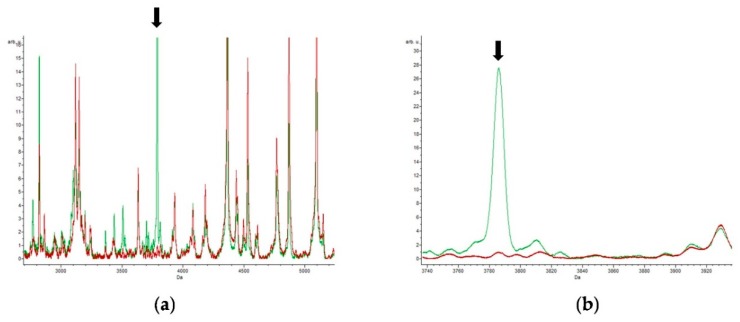
Ciprofloxacin antibiotic: (**a**) spectrum obtained in the presence of ciprofloxacin (green) and without antibiotic (red); (**b**) a detail of the main peak of interest at 3785.80 *m*/*z*.

A total of 25 peaks were detected in protein extracts from *E. coli* strains grown in the presence of ciprofloxacin. One of these peaks, the 6312.78 *m*/*z* peak, was highlighted by all three classification algorithms. This peak was also identified in the control samples, but at a lesser intensity. Three other peaks were found with at least two of the three algorithms. There was strong expression of proteins in the range of 3000 to 4000 *m*/*z*, and the masses 2359.22 *m*/*z*, 3432.73 *m*/*z*, 3503.91 *m*/*z*, 3698.04 *m*/*z*, 3785.80 *m*/*z*, and 5791.25 *m*/*z* only appeared in samples from bacteria grown in the presence of ciprofloxacin. Of all the antibiotics studied, ciprofloxacin was the only one that did not induce a spectrum with peaks similar to those induced by other antibiotics, which had higher peptide masses. The peak at 3785.8 *m*/*z* is illustrated in Figure 6.

For the aminoglycoside class of antibiotics, it was not possible to carry out the MALDI-TOF MS study, because there were not enough isolates with this type of resistance for a statistically sound analysis.

*E. coli* strains susceptible to polymyxin have been reported to be the negative mass spectrum found between 1600 to 2200 Da [40]. Although this study did not include the latest generation of antibiotics, such as polymyxins, daptomycin, or glycylcyclines, there have been studies already focused on some of these antibiotics, namely polymyxins in Gram-negatives. When compared to our control samples (without the addition of antibiotics), we founded that we had very residual peaks in that range. Therefore, compared to this study, our samples may be susceptible to this antibiotic. The authors also showed that in all strains of *E. coli* resistant to polymyxin, an additional peak of 1919.20 Da was observed regardless of the resistance mechanism involved (encoded by the chromosome or plasmid) [40]. In our study, this peak was not detected in any strain, proving once again that the strains are sensitive to this antibiotic. Regardless, further studies are needed for this antibiotic, because no experiment with polymyxin has been carried out, and the conclusions drawn in this work are preliminary, and only by comparison with other studies.

## 4. Conclusions

The present study brings a new perspective on antibiotic resistance and the normal functioning of the bacterial cell by obtaining more detailed proteomic information about resistances. Few studies have previously used MALDI-TOF MS for the detection of peaks that are specific to exposure to individual antibiotics. Despite being a preliminary study in the detection of putative protein biomarkers, the MALDI-TOF MS technique is here shown to have a high potential in the identification of potential biomarkers for the resistance responses to different antibiotics, especially for trimethoprim–sulfamethoxazole and chloramphenicol, in *E. coli*. This will pave the way for future research on the prevalence of antibiotic-resistant bacteria in various animals, focusing on the direct and indirect effects of these bacteria in our ecosystem and the evaluation of long-term effects on the animal and human population. MALDI-TOF MS, in combination with the processing and statistical analysis of the mass spectrum obtained for the different antibiotics, may contribute to a technique of identification for routine bacterial multidrug resistance.

It is expected that with the advent of this type of technology, the identification of specific mechanisms of resistance will be facilitated to improve and accelerate patient diagnosis and treatment. To go beyond the simple observation of mass peaks of interest, the corresponding proteins will have to be identified to better understand the fundamental pathways of antibiotic resistance.

## Figures and Tables

**Table 1:** Data on the origins of the *E. coli* strains used in this study.

Animal	Number of Animal Samples Collected	Type of Samples	Period of Collected	Geographic Location of Collection
Pigs	71	Faecal samples	September 2008 to March 2009	Slaughterhouse located in central Portugal
Iberian Lynx	128	Faecal samples	2008 to 2010	Doniga National Park, Sierra Morena, and Southern Spain
*Buteo buteo*	33	Faecal samples	September 2007 to February 2008	Pêneda do Gerês Natural Park or others conservation areas of Portugal
Birds of prey	119	Faecal samples	April to July de 2008	Center for Ecology, Recovery and Wildlife Surveillance
Boar	77	Faecal samples	December 2005 to February 2007	North of Portugal
Chicken	22	Different parts of a chicken	September to December 2007	Supermarket in Vila Real

**Table 2:** Phenotype and resistance genotype of *E. coli* isolates.

Animals	*E. coli* Isolates	Resistance Phenotype	Resistance Genes
Chicken breast	P6A	STR CN SXT CIP	*bla_TEM-52_aadA* aac(3)-II *sul2*
Chicken wings	A3A	TET SXT	*bla_CTX-M-1_ tet*(B) *sul2*
	A4A	AMP	*bla_TEM-52_ tet*(B)
Chicken gizzard	M1A	CN SXT CHL	*bla_CTX-M-1_*_4_*cmlA* aac(3)-II *sul3*
Chicken skin	Pe4A	TET SXT	*bla_TEM-52_ tet*(A) *sul sul2*
Birds of prey	13 103	AMP CIP TET K SXT	*bla_TEM_ bla_CTX-M-3_ tet*(A) *sul2*
	1 102	AMP CIP TET K STR SXT	*bla*_CTX-M-3_*tet*(A) *sul2*
	2 101	AMP CIP TET K SXT	*bla_TEM_bla*_CTX-M-3_*tet*(A) *sul2*
Boar	J31	AMP CIP STR SXT	*bla* *_TEM_* *bla*_CTX-M-3_ *sul2 sul3*
Pigs	SU50	TET CHL	*tet*(A) *cmlAbla*_CTX-M-1_
	SU54B	TET SXT CHL	*sul3 tet*(A) *tet*(B) *cmlAbla*_CTX-M-1_
	SU54C	TET SXT CHL	*sul3 tet*(A) *tet*(B) *cmlA bla*_CTX-M-1_
	SU62	TET	*tet*(A) *bla*_SHV-12_
	SU65	TET	*tet*(B) *bla*_CTX-M-1_
	SU80	TET SXT	*sul1 sul2 sul3 tet*(A) *bla*_CTX-M-1_
Lynx	L16	TET STR	*tet*(A) *aadA bla*_CTX-M-14_
	L98	STR SXT	*sul3 aadA bla*_SHV-12_
*Buteo buteo*	BU10A	AMP TET SXT	*sul1 sul2 sul3 tet*(A) *bla_TEM-1_ bla*_CTX-M-32_
	BU10B	AMP FOX STR TET SXT	*aadA ampC sul1 sul2 sul3 tet*(A) *bla_TEM-1_bla*_CTX-M-1_
	BU22A	TET STR SXT AMP	*aadA sul1 sul2 sul3 tet*(A)*bla_TEM-1_ bla*_CTX-M-1_
	BU32	STR	*aadA bla_TEM-1_ bla*_CTX-M-1_
	BU41A	TET SXT AMP	*sul1 sul2 aadA bla* *_TEM-1_* *bla*_CTX-M-1_
Human	CNR695	FOX CIP CHL SXT AMP	*bla*_CTX-M-14_
	CNR2630	FOX CIP CHL SXT AMP	*bla*_SHV-12_
	CNR1890	FOX CIP CHL SXT AMP	*bla*_CTX-M-1_
	CNR681	FOX CIP CHL SXT AMP	*bla*_CTX-M-9_
	CNR742	FOX CIP CHL SXT AMP TET	*bla*_CTX-M-14_
	CNR477	FOX CIP CHL SXT AMP TET	*bla*_CTX-M-14_
	BLSE176	FOX CIP CHL SXT AMP	*bla*_CTX-M-32_
	BLSE119	FOX CIP CHL SXT AMP TET	*bla*_CTX-M-32_

TET: tetracycline; AMP: ampicillin; CIP: ciprofloxacin; SXR, trimethoprim: sulfamethoxazole; K: kanamycin; CN: gentamicin; CHL: chloramphenicol; FOX: cefoxitin; STR: streptomycin.
